# Stable, fragile and recreated – a qualitative study of agency in everyday life with breast and prostate cancer

**DOI:** 10.1080/17482631.2019.1690391

**Published:** 2019-11-20

**Authors:** Suvi Holmberg

**Affiliations:** Faculty of Social Sciences, Tampere University, Tampere, Finland

**Keywords:** Agency, breast and prostate cancer, daily activities and habits, everyday life

## Abstract

**Purpose**: This study aims to explore how agency is constructed in everyday life with cancer in relation to daily activities and habits. Agency is approached as a key element of daily life existence, and it is constructed in terms of “acting in the world”, self-behaviour, changing routines, identity expectations and life course.

**Methods**: The study is based on a social constructionist approach and the data of 32 participants were gathered through a public call for narratives on “everyday life with breast and prostate cancer” in Finland in 2009. The analysis was conducted by utilizing a discursive research approach and coding.

**Results**: Three categories of agency were identified: *stable*—where agency continues fluently after cancer; *fragile*—where the ability to take care of daily activities has deteriorated; and *recreated*—where living with cancer adapts or creates a new basis for daily living.

**Conclusions**: The findings of the study suggest that everyday life activities and habits define and (de)construct agency, and that these constructions are tightly linked to the ill person’s overall life situation, physical abilities and cultural context. Having cancer can create new challenges to agency in daily life but does not suppress agency.

## Introduction

Statistics relating to the survival rates of cancer patients in the period 2014–2016 in Finland show that 91% of women with breast cancer and 93% of men with prostate cancer were still alive five years after their diagnosis (Finnish Cancer Registry, 2018). These high survival rates support the fact that people diagnosed with cancer live the majority of their lives *outside* of the medical or health care environment. This creates the need to study cancer in patients’ ordinary life contexts (e.g., Isaksson, Salander, Lilliehorn, & Laurell, 2016; Salander, Lilliehorn, Hamperg, & Kero, 2011). Previous research has focused on patients’ quality of life and everyday life experiences after cancer treatments (e.g., Kerr, Ross, Jaques, & Cunningham-Burley, 2018; Pedresen, Koktved, & Nielsen, 2013; Salander, Bergenheim, & Henriksson, 2000; Sekse, Raaheim, Blaaka, & Gjengedal, 2010). The experience of cancer brings profound changes in bodily and psychosocial functioning into patients’ day-to-day lives, and this generates the need for information and support from health care professionals (Pedresen et al., 2013; Sekse et al., 2010; Swenne, Jangland, & Arakelian, 2017). The side effects of treatment are recognized in the busy healthcare system, but schedule pressures and short patient encounters can make it challenging to formulate a clear rehabilitation plan or to take into account the other needs in patients’ everyday lives (Pedresen et al., 2013; Swenne et al., 2017). Because of the controversy over what patients get and what they actually need, it is important to study their everyday lives alongside the medical setting.

According to Felski (2000, p. 77–98), everyday life expresses itself through repetition, in a variety of spaces and in semi-automatic, distracted or involuntary habits. These elements are linked to cultural issues, and they influence people’s attitudes and social positions in life. Here, the focus is on the daily activities and habits that are accomplished and repeated in everyday life in various situations and spaces. People wake up, make coffee, take a shower, kiss their spouse goodbye and drive to work. In a healthy life, the daily routine can feel meaningless, but in a life with serious illness, these routines can act as an anchor on which one can rely when subjected to stressful situations (Salander, 2016, p. 347).

Studies show how cancer impacts all areas of everyday life: social relationships, work, routines, hobbies and the emotional and physical aspects of life. Isaksson et al. (2016) and Salander et al. (2011) argue that the actual impact depends on the individual patient’s personal coping characteristics and life circumstances. This means, for example, how former challenges in a person’s life can help them to cope with their cancer, or how facing unemployment can create additional pressures in daily life (Isaksson et al., 2016). From this viewpoint, dealing with cancer can be seen as an individual transition in the everyday life context (Isaksson et al., 2016; Salander et al., 2011). Despite the fact that cancer can dramatically change a person’s life, patients are eager to return to their everyday lives (La Cour, Johannessen, & Josephsson, 2009; Salander et al., 2000; Salander & Lilliehorn, 2016).

What could further enrich the current research is an analysis of the mundane acts of everyday life in relation to cancer (e.g., La Cour et al., 2009). In this study, daily activities and habits are defined as the concrete elements that capture the reality of living with cancer. Scrutinizing the meanings of ordinary daily activities and habits, aim to reveal how people respond to living with cancer. This perspective may help to narrow the gap between the busy health care system and individual life circumstances. This study contributes to the discussion from the perspectives of agency.

Here, agency is approached as a diverse and temporal concept connected with the entirety of habits, relations, feelings and values that are linked to interactions and the cultural context in which a person lives (Emirbayer & Mische, 1998; Hitlin & Elder, 2007; Honkasalo, 2009). Agency is understood as a key element of being in the world: people construct their existence in relation to their “acting-in-the-world” (Honkasalo, 2009, p. 58). Daily “acting” is influenced by the requirements of interaction, temporal orientation and situational circumstances, which imply different variants of agency (Hitlin & Elder, 2007). In Hitlin and Elder’s (2007) analysis, agency is expressed in four overlapping types: the existential, which refers to the human’s existential capacity for initiating and controlling self-behaviour; the pragmatic, which translates into the ability to innovate when familiar routines break down; identity, which expresses the capacity to act within socially prescribed role expectations; and life course, which refers to a retrospective analysis of decisions made at various turning points and transitions in life (Hitlin & Elder, 2007, p. 175–176). In the context of this study, an analysis of agency offers a way of getting closer to the realities of daily life with cancer: how people with breast and prostate cancer use their daily activities and habits to control their self-behaviour, how they relate to changing routines and identity expectations, or how they handle their cancer as part of their life course.

In previous qualitative cancer research, cancer has been seen to impact agency in many ways. In a study of women living with a genetic risk of breast/ovarian cancer, health in daily life was transformed into a project to be pursued, to the extent that the health project enhanced, constrained and questioned the women’s agency (Caiata-Zufferey 2015). Having cancer can also change the sense of agency, that is, how people act to regain control over their health and the valued aspects of their lives (Davies, Kelly, & Hannigan, 2018; Xuereb & Dunlop, 2003). From the perspective of ordinary daily activities such as eating, scrutinizing the nature of agency unveils complex links between social relationships, interactions and the need for credible information (Kassianos, Coyle, & Raats, 2015; La Cour et al., 2009). The relationship between agency and daily life with cancer has also been scrutinized in the context of decision-making (Davies et al., 2018) and from the viewpoint of activity and meaning-making in the daily lives of people with advanced cancer (La Cour et al., 2009). This study contributes to the discussion by exploring the meanings of daily activities and habits on agency and coping after a cancer diagnosis and it emphasizes the importance of ordinary “acting in the world” as a meaningful part of the cancer care process. In addition, this study presents the daily life of living with cancer from the perspectives of people whose time of falling ill, the state of their cancer and treatments they have had vary greatly. The specific research question asks, *How is agency constructed in everyday life with breast and prostate cancer in relation to daily activities and habits?*

## Methods

This study is based on the social constructionist approach (e.g., Burr, 2015; Conrad & Baker 2010; Potter, 1996). The framework proposes that living with cancer is not just a pathological issue, but rather, it is constructed in the social encounters of everyday life. Social encounters, in turn, are influenced by historical and cultural norms and by the economic structures of society (Burr, 2015; Conrad & Barker, 2010). The implications of having cancer are negotiated in a particular cultural and social context. This affects the way in which people experience illness and construct meaning around it (e.g., Charmaz, 2006). Providing a key position from which to scrutinize agency construction is language and the ways in which people use it (e.g., Burr, 2015).

### Setting and participants

The data were gathered in 2009 through a public call for written narratives of “everyday life with breast and prostate cancer” and the call was directed at people diagnosed with cancer and their loved ones. The call for narratives was published on Finnish cancer and patient organizations’ websites and in journals and on rehabilitation courses that targeted cancer patients and their loved ones. Respondents were requested to describe their everyday lives after their cancer diagnosis (work, free time, social relationships, thoughts and feelings about the body, sexuality and illness). They were asked to state the type of cancer, the time when they fell ill, how the illness had been treated and the current status of their illness. The researcher did not meet the participants and did not know who they were. This method of data collection is complementary to the methodology traditionally used in qualitative cancer research, which often uses interviews.

In total, 37 people decided to participate in the study. Of the participants, 21 wrote about breast cancer and 11 about prostate cancer. Five of the responses were written by loved ones, but these are not included in the study data for this article. The participants varied in age, marital status, employment status, time of falling ill and the type of cancer treatment they had received. The participants had all fallen ill between 1985 and 2009. The detailed characteristics of the participants are presented in Table I. The narratives varied between 1 and 24 pages (154–4523 words) in length, except for one text that consisted of 67 pages^1^ (18,327 words). Excluding the longest text, the average length of the breast cancer narratives was five pages (1178 words), and for prostate cancer it was two pages (545 words). In total, the data amounted to 202 pages (48,517 words). The data examples used in the results section were translated from Finnish into English. All personal details and identifying data were removed, and the names used in the examples are pseudonyms.

**Table I.** Characteristics of the 32 breast and prostate cancer participants^2^.

Characteristics	Women with breast cancer (*N* = 21)	Men with prostate cancer (*N* = 11)
**AGE IN YEARS**		
Mean Range	57.5 37–76	66 58–86
**Timeline between getting ill and writing the narrative** Range of the year of getting ill Range of the gap between getting ill and the time of participating in the study (in years) 0–3 years from getting ill 4–7 years from getting ill More than 8 years from getting ill	1985–2009 0–24 14 (66.7%) 4 (19.0%) 3 (14.3%)	1995–2008 1–14 4 (36.4%) 5 (45.4%) 2 (18.2%)
**Marital status** Intimate relationship Single Widow Unknown	13 (61.9%) 2 (9.5%) 4 (19.1%) 2 (9.5%)	10 (91.0%) 1 (9.0%)
**Employment status** Employed Unemployed Pensioner Unknown	11 (52.4%) 2 (9.5%) 8 (38.1%) -	2 (18.2%) 1 (9.0%) 4 (36.4%) 4 (36.4%)
**Treatments received^3^** Surgery Chemotherapy Radiotherapy Hormones Incurable cancer	21 (100.0%) 11 (52.4%) 9 (42.9%) 7 (33.3%) 2 (9.5%)	6 (54.5%) - 8 (72.7%) 5 (45.5%) 3 (27.3%)

### Ethics

The research was not conducted for any specific cancer-related institution, and responses were accepted from any private person who saw the call for narratives and wanted to contribute to the study. Any detailed personal information was not systematically collected and participants were able to use aliases. The guidelines for the call for narratives declared clearly how the texts would be used and gave a guarantee of anonymity and confidentiality. Any other person than researcher did not have access to the raw data. Participants decided and evaluated for themselves what to write. The researcher’s contact information was made available for any further questions or withdrawal from the research. With regard to ethical issues, all the instructions in the ethical guidelines of the Finnish Advisory Board on Research Integrity (2009, 2012) and World Medical Association declaration of Helsinki (2013) were precisely followed.

### Analysis

The analysis was built on a discursive research practice that emphasizes respondents’ accounts and how they construct their world through their talk and texts (e.g., Charmaz, 2006; Potter, 1996). The analysis focused on how people describe their agency in relation to their daily activities. Systematic comparisons between women’s and men’s agency constructions were out of the scope of the analysis, but gender similarities and differences concerning participants’ everyday life accounts have been considered elsewhere (see Holmberg, 2017).

The preliminary analysis concentrated on *what* kind of daily activities and habits were described in the data. The next phase of the analysis focused on the categories of agency, and specifically on how agency was constructed in relation to daily activities connected to self-behaviour, changing routines, identity expectations and life course in the everyday lives of those with cancer. The analysis followed the coding process described by Gibbs (2007) and utilized the qualitative software ATLAS.ti. The concrete phases were:

1. *Categorizing daily activities and habits*. At first, all narratives were carefully read through and the data were imported into ATLAS.ti, which was used to organize and code the texts. The descriptive coding highlighted all phrases that interpreted everyday activity and habits. Then, individual codes were combined to create eight broad categories.
2. *Categorizing agency*. Second, the coded phrases were reconsidered in relation to eight daily activity and habit categories. The focus of the analysis was now on how the participants related to their daily activities and habits and how they used them to construct their own agency in relation to self-behaviour, daily routine changes, identity expectations and life course. The data were recoded, and eight categories of agency were identified. Agency categories were not clearly separated sets but they overlapped and seemed to divide between agency that either supported or prevented managing in daily life with cancer.
3. *Reorganizing agency categories*. The analysis continued by reconsidering and reorganizing the agency categories through a focus on supportive and preventive forms of agency in daily life. Agency was defined supportive, if daily activities and habits produced continuality in daily life and preventive, if they seemed to challenge maintaining agency. During the reorganizing process also the stable form of agency stood out, which meant that the agency seemed to locate between supportive and preventive forms. The analysis continued by summing up the supportive, stable and preventive elements from eight agency categories. Finally, three central agency categories emerged: *stable, fragile and recreated agency*.

The steps of the analysis process are presented in Tables II and III.

**Table II.** First two steps of the analysis process of 32 breast and prostate cancer narratives.

Analysis steps	Example	Full list of categories
1. Categorization of daily activities and habits	“At first, *radiation therapy* went without *side effects*, until after the 22nd time I felt *vexation in my rectum* and a compulsory need to *urinate*”. Categories that emerge from the example: Regular cancer treatments and control check-ups Physicality	Economic issues Hobbies and leisure time Physicality Regular cancer treatments and control check-ups Routine homework and daily chores Sexual intimacy Social participation Working life
2. Categorization of agency	“I was *working* as a receptionist, 44 years old single parent, healthy woman, when *one phone call transformed me from a nurse to a patient. Changed me for the rest of my life”*. Agency category that emerges from the example: Altered agency	Active agency Altered agency Physical agency Recovering agency Resilient agency Suffocating agency Surviving agency Uncertain agency

**Table III.** Third step of the analysis process of 32 breast and prostate cancer narratives.

Full list of categories of agency	Reorganization	Identified final categories of agency
a) Active agency b) Altered agency c) Physical agency d) Recovering agency e) Resilient agency f) Suffocating agency g) Surviving agency h) Uncertain agency	Supportive continuance of agency: ● a, c, d, e, g Stable continuance of agency: ● a, b, d, e, g Preventive continuance of agency: ● b, c, e, f, h	Stable: a, b, c, d, g Fragile: b, c, e, f, h Recreated: a, b, c, d, e, g, h

## Results

### Stable agency

In this category, agency is influenced by the cancer, but it still continues fluently. This means that daily activities and habits continue despite cancer. Pauli, an unemployed man in his 60s who was living with his spouse, was treated with hormones and radiotherapy a year before writing his narrative. He describes the onset of his prostate cancer:

After I received the letter of diagnosis, for a couple of days I felt my life was over. It was, right now, the end of my life. However, as humans, we possess a strong defence system, which, after activation, starts to organize things into the right order. For me, the fighting spirit became so strong that I actually thought the illness was quite insignificant before I received the treatment. There were days when I didn’t even remember that I had cancer. Hobbies and other things that I liked to do helped me to forget the illness.

In Pauli’s description, as in many other accounts, everyday life and agency seem to halt for a moment after the diagnosis of cancer, but they recover quickly. Agency is constructed as being stable due to the personal trait called “a fighting spirit”, which helps Pauli to “organize things into the right order”. This verifies how daily activities, “hobbies and things I liked to do”, are defined as being more significant than the cancer. Pauli is able to take control over the cancer in his everyday life with the help of daily activities and by maintaining stable agency.

Stable agency can also be constructed when the illness process does not proceed fluently. Emma, a woman in her 50s who lived with her spouse and was working, fell ill five years before writing the narrative and had been treated with surgery, chemotherapy and radiotherapy. She had to wait quite a long time before the breast cancer surgery took place:

So I had to wait two more months for the surgery and wasn’t operated on until the beginning of May. I kept on working until the day before the surgery. Everybody wondered how I managed, but to me work was like therapy.

Here, it seems that daily activity, “working”, helps to relieve living with cancer. But, as Emma later continues, at the same time it can have ambivalent meanings:

At the time, I was very tired at work, and I remember that my first thought after diagnosis was ‘great, I can leave with a clear conscience to be ill at home’ […] We travelled a lot during my illness, as there were two weeks between chemotherapy [sessions], and I was in quite good shape.

Emma’s description unveils the burden of daily routines: “at the time I was very tired at work”. Having cancer appears to enable her to detach herself from tiring work and to replace it with other meaningful actions, such as travelling. Agency is constructed as being stable despite the cancer. From these examples, it would seem that constructing stable agency does not depend very much on employment status or fluency of the treatment process but rather on the possibility of making meaningful choices in relation to daily activities and habits.

### Fragile agency

In this category, agency becomes fragile because of the cancer. This means that participants do not manage to take care of their daily activities and habits in the way they are accustomed to. The process can last for varying timespans: from the mere duration of the active cancer treatment to several years after falling ill. In this study, women with breast cancer constructed fragile agency when they interpreted the changes in their daily functioning in ways that enabled them to accomplish their daily chores. Laura, a woman in her early 40s, who fell ill three years before writing the narrative and was living with her spouse and two small children, reflects on her daily life during chemotherapy and radiotherapy treatments:

There is a lot of housework to be done in a family with children, and this demands time, organisation and strength. Often domestic issues are mainly the mother’s responsibility, so if the mother/woman falls ill, the need for external help becomes considerably greater. Surgery and treatments consume all the strength of a patient, and she can’t act like a responsible parent or worker. In our family, all practical chores were left to my husband to take care of because our grandparents lived so far away, and my mother was taking care of her own aged parents.

Agency becomes fragile because Laura cannot take care of the daily chores as she used to. Daily activities are greatly gendered when “domestic issues” are expected to be the “responsibility” and obligation of the “mother” or “woman”. This can be interpreted as indicating that her identity as a mother became threatened. Laura also defines herself as a “patient”, which culturally emphasizes her changed agency from being active to more passive. This alleviates the pressure on her and justifies her constructing a fragile agency. In a similar manner, Maria, a retired widow in her 60s who was treated with surgery and radiotherapy and who had lived with breast cancer for 24 years before writing her narrative illustrates her working life and her fragile agency:

Then, in summer 1987 my back became sore. I was still working in the office when the occupational doctor prescribed 10 heat massage treatments. It didn’t help. At the end of the year, after hearing about my back problems, the occupational doctor contacted the oncologists. This led to a referral for a skeletal map, and as a Christmas present I received news of the metastasis. The year 1988 began with radiotherapy. After the first treatment, the pain was significantly relieved. But the metastasis in my lower back still caused me pain, and by the end of the year I had to retire on a disability pension, in my 40s.

In this description, issues relating to physical distress construct fragile agency. Maria reflects on her progressive back problems that finally turned out to be “news of the metastasis”. Everyday life was coloured with “pain” that reduced the possibility of taking care of daily routines. By the end, she “had to retire on a disability pension” at a relatively young age. Maria’s story is uncommon among the data. Despite the metastasis, she has lived with her breast cancer for another 24 years.

In addition to cancer’s effects on domestic responsibilities and working life, changes in sexuality and mobility maintain fragile agency. Henri, a man in his mid-60s living with his spouse, was treated with surgery, hormones and radiotherapy because of the spread of his cancer. He describes the long-lasting side-effects of the surgery:

Like the previous description [a long account of unsuccessful treatment and difficult side-effects] shows, I have had to forget all things linked to exercise. The length of my walks is determined by the need to change diapers, as I don’t have, at least for now, proper urinary control. My sexuality, or at least my physical ability, vanished with the surgery.

The physical side effects construct fragile agency. The daily activities of exercising, walking and sexual intercourse are difficult to maintain, and fragile agency is constructed in relation to “proper urinary control” and sexuality that “vanished with the surgery”. Despite this, Henri´s agency does not entirely come to an end. He still continues his walks even though “the length of my walks is determined by the need to change diapers”. Agency has become fragile, but it still exists. From these examples, it can be argued that the more cultural responsibilities and physical restrictions participants have in their lives the more pressure will be directed at their agency. However, agency does not vanish from everyday life, it is constructed through a resilient attitude and by coping with the physical side-effects.

### Recreated agency

In this category, living with cancer constructs a recreated agency in which participants are able to adapt and create a new basis for their daily lives. This manifests itself in situations where daily life begins to demand new ways of “being”. This can mean, for example, that fragile agency becomes a permanent way in which to continue living. Liisa, a widow in her 50s living on a disability pension, who was treated with surgery, chemotherapy and radiotherapy because of the spread of her cancer, describes her new way of living in her narrative she wrote four years after the treatments:

Now, life is about coping from day to day, but if God allows, I’ll carry on like this for as long as possible. After all, I can still enjoy life as it’s pleasing to wake up to a new day.

Liisa describes how her life is about “coping from day to day”. Recreated agency is constructed as a position between fragile and stable agencies when she reflects on the nature of her life and the possibility of its continuing as before. The daily habits of “waking up” and “coping” construct a recreated agency in a relationship with time that seems ambiguous. Simultaneously, the temporality of everyday life is defined as hard to manage, but it is also defined as an element that maintains agency. Recreated agency can also mean changing the ways in which daily activities and habits are maintained or controlled. Anna, a retired woman in her 60s who was treated with surgery, chemotherapy and radiotherapy 17 years before writing her narrative, describes a recreated agency in which, while being a single mother, she has found the courage to study for a new profession:

Is this my work for the rest of my life, and when my other daughter leaves, what should I do here, could I do something else? My oldest daughter came up with a great supportive idea—start studying. And that’s what I did. I studied a new artisan occupation that I had dreamed of doing since I was young. Later, I also moved and changed my workplace: I had a challenging new job that required applying the skills of two occupations at the same time. I also settled in a new relationship. I argue that without the cancer I probably wouldn’t have begun studying to achieve my dreams.

The experience of cancer has encouraged Anna to “follow her dreams” and has helped her to make big changes in her life. Recreated agency appears to be something that has improved the quality of her life, but it would not have happened without the cancer. At the same time, future changes in her family situation, namely, “when my other daughter leaves”, support constructing recreated agency and help to make a suitable space for it in daily routines. As a part of her life course, having cancer seems to represent an almost positive turning point. However, constructing recreated agency does not mean that everyday life has changed for the better in all the narratives. Mika, a man in his mid-60s and living with a spouse, has suffered from prostate cancer for 11 years and was treated with surgery, chemotherapy and hormones. He describes his daily life:

You just have to live with it, but every time you hear the results of blood tests it is frightening. After my sick leave, I continued with my hobbies as usual. I swim, cycle, ski and do Nordic walking. I go to monthly peer-group meetings and weekly meetings at the Cancer Association.

During the last 11 years, Mika has learned to live with his cancer. “Monthly and weekly meetings” construct a recreated agency, which seems quite stable when linked, as it is, to his hobbies, peer-support and other activities. At the same time, his agency is constructed as fragile because there seems to be a continuing ambivalence in his everyday life due to the “blood tests” that cause fear and uncertainty. This makes his recreated agency vulnerable in daily life. Mika’s account shows the extent to which agency categories can overlap. From these examples, it can be argued that constructing agency is not a steady process, but instead, it is continuously changing and depends on the activities and circumstances of one’s life. In addition, the passage of time between falling ill and the present does not mean that agency has automatically become either recreated or stable, it can be constructed as fragile even years after falling ill.

## Discussion

This study approaches falling ill with breast or prostate cancer from the perspective of agency in relation to everyday life activities and habits. The results show how cancer can influence everyday life in different ways after falling ill. This resonates with previous follow-up studies that focus on different cancer types (Isaksson et al., 2016; Salander et al., 2011; Swenne et al., 2017). As the results of the study show, agency in everyday life is not a static phenomenon but instead, is tightly linked to the life situation of the individual. Here, the everyday activities were in the central position as participants evaluated the impacts that their cancer had had and how they had adapted to the changes caused by the cancer. Daily activities and habits defined and (de)constructed their agency. In this study, marital or employment status were not considered significant when constructing agency, but the possibility of freely choosing one’s own daily activities was. Daily routines seemed to help in seeing the cancer as “a passed parenthesis”, which in turn, helps in the regaining of ordinary life (Isaksson et al., 2016).

At the same time, the study shows how agency can be constructed as fragile if the ability to take care of daily activities has deteriorated. This challenges the idea that everyday life is “an anchor one can rely on” (Salander, 2016, p. 247) and shows how daily activities can also be seen as elements that deconstruct agency and threaten identity in daily life. From this perspective, daily routines are not just indifferent necessities, but they are coloured with cultural meaning, which has a lot of power in defining the actions of everyday life (Felski, 2000, p. 77–98).

This study shows that agency can also be constructed as recreated if daily life begins to demand new ways of being. In some examples, the cancer seemed to offer a positive way of starting to move towards new possibilities in a dissatisfying life situation and thus helped the individual to respond to cancer as a part of their life course (see also Salander et al., 2011). Alternatively, it could mean continuing with everyday life but accepting physical restrictions and uncertainty. What is common to both of these cases is that having cancer can challenge agency, *but it does not suppress it*. This is in line with the findings of Faircloth, Boylstein, Rittmann, Young, and Gubrium (2004), where individuals recovering from stroke were seen as “agentic subjects”, who were working actively towards recovering a certain quality of life. Life with cancer may not stay the same, but the majority of patients are able to go on living despite the uncertainty or the threat to life (La Cour et al., 2009; Salander et al., 2000; Salander & Lilliehorn, 2016). Upholding stable, fragile or recreated agency also upholds everyday life existence.

Honkasalo (2009) uses the term “small agency” when referring to actions in uncertain and difficult everyday life circumstances due to heart disease. Small agency exists in situations where there is nothing else to do but to “endure” and take one day at a time (Honkasalo, 2009). In this, study agency is formed somewhat similarly, and the results are complementary in the sense that small agency is not dependent on the chronic nature of the illness, but it can be constructed during diverse periods of the illness process. Continuing with everyday routines and activities offers a feeling of safety and a way of maintaining a sense of control and normal life even if it is a struggle (La Cour et al., 2009; Pedresen et al., 2013; Salander & Lilliehorn, 2016; Xuereb & Dunlop, 2003). The repetition of habits in daily life may also be comforting because it implicitly generates a sense of eternity (Salander et al., 2000), or, as La Cour et al. (2009, p. 474) put it, “routines and continuity that seem to be associated with order and security amid the humdrum of everyday life while providing a platform for agency”. It is possible to argue that the continuity of even a small agency helps patients to normalize the illness as part of their life and biography (Faircloth et al., 2004).

The time between the participants falling ill and writing their narratives varied between 0 and 24 years (see Table I). This shows not only the fact that having cancer is a significant part of individual life course but that it also has long-lasting impacts on the constructions of agency in everyday life. Agency requires temporal orientation, and as Hitlin and Elder (2007, p. 175) express it: “Agentic actions involve differential orientation towards present and future. Temporal orientations can be analytically separated and implicate different aspects of the self within action. Individuals shift their time horizons based on the problems that emerge within situated interaction.” Here agency seems to be constructed fairly similarly between the past, the present and the future, despite the differences in the length of time between participants falling ill and writing their narratives. More notably, the differences stem from the differing life situations of the participants. However, the temporality of everyday life seems to give the participants possibility of constructing and evaluating their agency in relation to the past, present and future.

### Limitations of the study

The data set does not allow for broad generalizations. The results should not be approached as presenting “facts” but as real-life insights that reveal valuable information about the social realities of living with cancer. Another limitation of the study relates to the data gathering method. It can be argued that a public call for narratives was not a very effective way to gather data as only 37 participants responded. Still, it is worth remembering that in the year 2009 the possibilities for “publishing” the call were limited: for example, people did not use social media in the same way they do today. In addition, the requirement to produce written data may have deterred some people from responding if they were not skilled in expressing themselves in writing or were too ill to write. The data, therefore, include only ably written accounts, but some of the accounts were “more akin to outpourings of anguish” (Grinyer, 2004, p. 1328). There was no way to assess the extent to which the patients’ condition was a factor in determining their participation. However, the accounts clearly described physical and emotional exhaustion during different stages of the illness. The data gathering method can also be criticized for not giving the researcher the possibility of getting clarifications concerning the narratives. Nevertheless, in the present study the focus is on the language that the participants used when describing their everyday life and agency, and not on them as individuals (e.g., Burr, 2015).

Finally, the research data were collected in 2009, and this could draw criticism. However, in the light of the detailed analysis and the insights from previous literature, it is possible to argue that constructions of agency in everyday life with cancer have not changed significantly between 2009 and 2019. For example, during the past decade there have been many biomedical advances in cancer care, but a critical review of sociological research in the period 2007–2017 points out that biomedical knowledge has also brought new challenges regarding the ways in which people experience cancer, their identities and their responsibilities in their daily lives (Kerr et al., 2018; see also Paal, 2010, p. 37–40). This supports the argument that the ways in which cancer affects peoples’ everyday lives and the constructions and fluctuations in agency are not bound to any particular era (e.g., Caiata-Zufferey 2015; Davies et al., 2018).

## Conclusions

In conclusion, this study expresses how ordinary life plays a significant role in the illness process. This means that the “voice of the life-world” (Salander et al., 2000; Salander & Lilliehorn, 2016), that is, the meaning of daily activities and agency, cannot be disregarded in the cancer care process. In the medical setting, the importance of agency construction should be recognized as an aspect of developing holistic care. For instance, by paying closer attention to the daily routines of a patient, health care personnel will be able to provide better support to patients who are no longer in the health care setting. Additional research on how health care personnel perceive the meaning of daily life as part of the cancer care process would be of great value.

The results of this study point to two main conclusions. First, daily activities and habits strongly define and (de)construct agency, and the constructions are intensely linked to patient’s overall life situation, physical abilities, cultural context and temporality. Second, having cancer can cause new challenges to agency in daily life, but it does not suppress agency. The continuance of agency does not depend on the prognosis of the illness. Patients keep constructing their agency in overlapping and diverse ways by drawing on their individual resources.

## Notes

1. The written form and delivery of the narratives varied: 19 narratives were delivered by email and 13 by mail. Six of the narratives were handwritten. Before analysis, all narratives were compiled in electronic form and anonymized. The data corpus was standardized by saving the narratives with the font Times New Roman, font size 12 and line spacing 1.5, except for the longest, a 67-page text, which was saved with single line spacing.

2. The percentages are calculated against the group totals. The values are presented to an accuracy of one decimal point due to the small divider.

3. Most participants received more than one form of treatment.
